# Bridging Therapy Versus Intravenous Thrombolysis Alone in Patients With Medium Vessel Occlusion Stroke: A Single‐Center Cohort Study

**DOI:** 10.1002/brb3.71671

**Published:** 2026-08-12

**Authors:** Di Hu, Mo Yang, Zhenghua Gu, Jie Fan, Lizhi Yu, Chengcai Xia, Qingguo Ren

**Affiliations:** ^1^ School of Medicine Southeast University Nanjing Jiangsu PR China; ^2^ Cerebrovascular Disease Center Nanjing Pukou People's Hospital (Liangjiang Hospital Southeast University) Pukou District, Nanjing China; ^3^ Department of Neurology Nanjing Pukou People's Hospital (Liangjiang Hospital Southeast University) Pukou District, Nanjing China; ^4^ Department of Neurology Zhongda Hospital Southeast University Nanjing PR China

**Keywords:** bridging therapy, endovascular thrombectomy, intravenous thrombolysis, medium vessel occlusion, stroke

## Abstract

**Background:**

To compare the outcomes of bridging therapy versus intravenous thrombolysis (IVT) alone in patients with medium vessel occlusion (MeVO).

**Methods:**

Consecutive MeVO patients between January 2019 and December 2024 were retrospectively included and classified into IVT alone or bridging therapy groups. The primary outcome was a favorable outcome, defined as a modified Rankin Scale score of 0–1 at 90 days. Secondary outcomes included functional independence (mRS 0–2), symptomatic intracranial hemorrhage (sICH). Inverse probability weighting (IPW) and IPW‐adjusted logistic regression were used to balance baseline covariates and assess treatment associations. Per‐protocol and as‐treated sensitivity analyses were performed to assess the robustness of the primary findings.

**Results:**

A total of 248 patients with MeVO treated with IVT were included, of whom 86 received bridging therapy. After IPW adjustment, there was no significant difference in the rate of favorable outcome between the bridging therapy and IVT alone groups (59.3% vs. 62.2%; adjusted odds ratio [aOR], 1.22; 95% confidence interval [CI], 0.75–1.99). Similarly, bridging therapy was not associated with improved functional independence (73.5% vs. 76.3%; aOR, 1.18; 95% CI, 0.66–2.12) or increased risk of sICH (8.0% vs. 5.9%; aOR, 1.37; 95% CI, 0.50–3.75). Both per‐protocol and as‐treated sensitivity analyses yielded similar results.

**Conclusions:**

Bridging therapy did not improve overall outcomes compared with IVT alone in MeVO patients.

## Graphical abstract

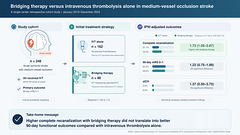

## 1 Introduction

While endovascular thrombectomy (EVT) is the standard of care for acute ischemic stroke secondary to large vessel occlusion (LVO), its role in medium vessel occlusion (MeVO) remains less clearly defined (Turc et al. 2023). Recent randomized trials—DISTAL, ESCAPE‐MeVO, and DISCOUNT—compared EVT plus best medical management (BMM) with BMM alone, yet none demonstrating a significant benefit of EVT (Psychogios et al. 2025; Goyal et al. 2025; Clarençon et al. 2024).

Several factors related to clinical and anatomical heterogeneity may underlie these discouraging findings. The enrolled cohorts included both proximal (e.g., second segment of the middle cerebral artery [M2 MCA], first segments of the anterior [A1 ACA] and posterior cerebral arteries [P1 PCA]) and distal MeVOs (e.g., M3–M4, A2–A3, P2–P3), which markedly differ in anatomical accessibility and the risk–benefit profile of EVT (Qiu et al. 2025). Stroke severity also varied considerably, with many patients presenting with mild deficits who may achieve favorable outcomes with BMM alone (Qiu et al. 2025; Qiu et al. 2025; Qin et al. 2023). Furthermore, a substantial number of patients in the BMM arms received intravenous thrombolysis (IVT), which may have led to effective recanalization in some cases—particularly in distal MeVOs where clot burden is lower and thrombolysis more effective (Seners et al. 2016). These heterogeneous factors may help explain the lack of clear benefit observed in unselected MeVO populations.

The present study aimed to compare clinical outcomes between bridging therapy and IVT alone among patients with MeVO stroke treated with IVT.

## 2 Material and Methods

### 2.1 Study Design and Population

We conducted a single‐center retrospective cohort study of consecutive patients with acute ischemic stroke due to MeVO between January 2019 and December 2024. Eligible patients presented within 24 h of symptom onset and received IVT as part of their initial management. MeVO was defined as an occlusion in the M2–M4 MCA, A1–A3 ACA, or P1–P3 PCA, confirmed on baseline computed tomography angiography (CTA) (Psychogios et al. 2025; Goyal et al. 2025; Clarençon et al. 2024). To reduce anatomic heterogeneity, M2 occlusions involving a dominant‐caliber branch supplying the majority of the MCA territory were excluded. Patients were excluded if they met any of the following criteria: (1) large vessel occlusion involving the intracranial internal carotid artery, M1 MCA, basilar artery, or dominant M2 branch; (2) prestroke modified Rankin Scale (mRS) score ≥2; (3) missing key baseline clinical or imaging data; and (4) lost to follow‐up or missing 90‐day mRS assessment. Due to the retrospective nature of this study, the Institutional ethics Committee waived the ethical application for this study. The study protocol followed the guidelines of the World Medical Association Declaration of Helsinki and was approved by the Ethics Committee of our institution. For this retrospective study, the informed consent was waived to participate by our ethics committee.

### 2.2 Treatment Allocation

Treatment groups were defined according to the initial treatment strategy at the time of IVT administration. Patients were stratified into two groups: (1) IVT alone group: Patients for whom no EVT was planned at the time of IVT administration. This group also included those who later underwent rescue EVT due to early neurological deterioration (END), as EVT was not part of their initial management plan. (2) Bridging therapy group: Patients who were selected for planned EVT following IVT as part of a bridging strategy. In our institution, EVT candidacy was determined by a multidisciplinary stroke team comprising neurologists, neurointerventionalists, and neuroradiologists, based on baseline National Institutes of Health Stroke Scale (NIHSS), anatomical occlusion site, perfusion imaging (Tmax >6 s, core/penumbra mismatch), vessel accessibility, and patient‐specific factors such as comorbidities or relative contraindications. The institutional protocol generally prioritized EVT for patients with proximal MeVOs (M2 MCA, A1 ACA, P1 PCA) who had either severe neurological deficits or substantial at‐risk tissue on perfusion imaging (Nogueira et al. 2024). In contrast, initial IVT alone was generally favored in patients with distal MeVOs (M3–M4, A2–A3, P2–P3), mild neurological deficits (NIHSS <6) without functionally limiting symptoms, and a small hypoperfused volume on perfusion imaging. For patients who did not clearly fit either typical profile, treatment allocation was determined on a case‐by‐case basis after multidisciplinary discussion and overall assessment of clinical severity, imaging findings, technical feasibility, and patient‐specific considerations. EVT could still be performed subsequently in initially IVT‐alone patients if END occurred or if reassessment by the treating team indicated that EVT was appropriate.

### 2.3 Thrombectomy Procedure

Endovascular procedures were performed under either groin‐level local anesthesia or conscious sedation, depending on the patient's clinical condition and the joint assessment of the neurointerventionalist and anesthesiologist. Mechanical thrombectomy was conducted using devices suitable for medium‐caliber intracranial vessels. Commonly used stent retrievers included the Solitaire AB (4 × 20 mm; Medtronic, Irvine, CA, USA), and EmboTrap II (3 × 22 mm or 4 × 22 mm; Cerenovus, Irvine, CA, USA). Aspiration techniques were applied either as a primary strategy or in combination with stent retrievers. Aspiration catheters included the SOFIA 5F (MicroVention, Aliso Viejo, CA, USA) and 3MAX/4MAX Reperfusion Catheter/ACE 60 (Penumbra Inc., Alameda, CA, USA). If reperfusion was deemed unsatisfactory after multiple thrombectomy attempts, rescue strategies were employed. These included intra‐arterial thrombolysis or intra‐arterial administration of glycoprotein IIb/IIIa inhibitors (e.g., tirofiban), at the discretion of the operator.

### 2.4 Outcomes Evaluation

END was defined as an increase of ≥4 points in the National Institutes of Health Stroke Scale score from baseline or ≥2 points in any single National Institutes of Health Stroke Scale item, within 24 h of IVT (Hu et al. 2025). Neuroimaging follow‐up was conducted according to a prespecified protocol. All patients underwent non‐contrast CT (NCCT) within 24 h after reperfusion therapy to evaluate for hemorrhagic transformation (HT). Symptomatic intracranial hemorrhage (sICH) was defined in accordance with the Heidelberg Bleeding Classification, which integrates both radiologic findings and clinical deterioration (Von Kummer et al. 2015). In patients treated with IVT alone, recanalization status was assessed using follow‐up vascular imaging with either CTA or MR angiography performed between 24 and 72 h post‐treatment. Recanalization was classified into three categories based on contrast opacification beyond the occlusion site: no recanalization (no contrast filling beyond the occlusion), partial recanalization (contrast filling in some distal branches), and complete recanalization (contrast filling in all distal branches) (Zaidat et al. 2013). In patients who underwent bridging therapy, reperfusion was evaluated using the MeVO‐A‐TICI grading system, defined as follows: grade 0, no reperfusion; grade 1, contrast penetration without or with only minimal distal filling; grade 2a, partial reperfusion with <50% distal territory filling; grade 2b, substantial reperfusion with 50%–90% filling; grade 2c, near‐complete reperfusion (90%–99%); and grade 3, complete reperfusion (100%) (Goyal et al. 2021). For consistency and comparison across groups, MeVO‐A‐TICI grades 0–1 were classified as no recanalization, grades 2a–2b as partial recanalization, and grades 2c–3 as complete recanalization. A subsequent NCCT or MRI was obtained on day 5 (±1 day) to estimate final infarct volume. Functional outcomes were assessed using 90‐day mRS, with favorable outcome defined as mRS 0–1. Structured mRS assessments were conducted via telephone by certified stroke nurses who had completed standardized mRS evaluation training.

### 2.5 Statistical Analysis

All statistical analyses were performed using R software (version 4.4.0). Continuous variables were expressed as mean ± standard deviation (SD) or median with interquartile range (IQR), and compared using the Student's *t*‐test or Mann–Whitney *U* test, as appropriate. Categorical variables were summarized as counts and percentages and compared using the chi‐square test or Fisher's exact test. The primary outcome was a favorable functional outcome, defined as mRS score of 0–1 at 90 days. Secondary outcomes included functional independence (mRS 0–2), and mortality (mRS 6). To adjust for baseline imbalances between the bridging therapy and IVT‐alone groups, Inverse probability weighting (IPW) based on a propensity score model was applied. All analyses were conducted in the original cohort of 248 participants (162 in the IVT‐alone group and 86 in the bridging therapy group), with no additional exclusions during the weighting procedure. IPW generated a weighted pseudo‐population for treatment effect estimation; weighted group sizes were considered estimates from the pseudo‐population rather than the number of observed participants. Propensity scores were estimated using logistic regression incorporating baseline covariates, including age, sex, NIHSS score, occlusion location (proximal vs. distal MeVOs), perfusion parameters (CBF <30% and Tmax >6 s volumes), and time from symptom onset to hospital admission. Covariate balance after weighting was assessed using standardized mean differences (SMDs), with values <0.1 generally indicating good balance. The treatment effect of bridging therapy versus IVT alone was then estimated using IPW‐weighted multivariable logistic regression models further adjusted for age, sex, baseline NIHSS score, occlusion location, perfusion parameters, time from symptom onset to admission, and recanalization status (complete vs. incomplete or none), in a doubly robust fashion. Effect estimates were expressed as odds ratios (ORs) with 95% confidence intervals (CIs) to quantify the association between treatment and outcomes.

To evaluate the impact of treatment crossover due to rescue EVT, we conducted two sensitivity analyses in the matching pre‐cohort population. In the as‐treated analysis, patients in the IVT‐alone group who subsequently underwent rescue EVT were reclassified into the bridging therapy group, reflecting the therapy actually received. In the per‐protocol analysis, these patients were excluded from the analysis to assess outcomes under strict adherence to the initial treatment plan. Outcome measures included favorable outcome (mRS 0–1), functional independence (mRS 0–2), and death (mRS 6) at 90 days. For each analysis, OR with 95% CI were calculated using multivariable logistic regression adjusted for baseline covariates (age, sex, baseline NIHSS score, occlusion site, perfusion parameters, and recanalization status).

## 3 Results

### 3.1 Study Population and Baseline Characteristics

**FIGURE 1:**
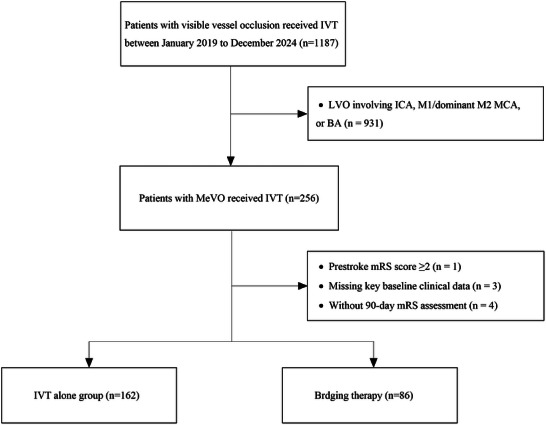
**Flowchart of patient selection**. IVT, intravenous thrombolysis; LVO, large vessel occlusion; ICA, internal carotid artery; M1 MCA, first segment of the middle cerebral artery; BA, basilar artery; MeVO, medium vessel occlusion; mRS, modified Rankin Scale.

A total of 248 patients with MeVO who received IVT, with or without subsequent EVT, between January 2019 and December 2024 were included (Figure 1). The mean age was 66.2 ± 11.0 years, and 152 patients (61.3%) were male. The median baseline NIHSS score was 4 (IQR, 2–7). Proximal MeVOs (M2, A1, P1) was identified in 118 patients (47.6%), while 130 patients (52.4%) had distal MeVOs. Among the total cohort, 86 patients (34.7%) underwent bridging therapy and 162 (65.3%) received IVT alone. As summarized in Table 1, patients in the bridging therapy group had significantly higher baseline NIHSS scores (median 6 vs. 3; *p* < 0.001), a greater proportion of proximal MeVOs (62.8% vs. 39.5%; *p* < 0.01), and larger Tmax >6s volumes (median 45 vs. 34 mL; *p* < 0.001). Complete or partial recanalization occurred in 84.9% of the bridging group (complete: 43.0%, partial: 41.9%) compared to 63.6% in the IVT‐alone group (complete: 25.9%, partial: 37.7%; *p* < 0.001). In the IVT‐alone group, complete recanalization was achieved in 6 of 45 patients (13.3%) with proximal MeVOs and in 36 of 117 patients (30.8%) with distal MeVOs. In the bridging therapy group, complete recanalization occurred in 31 of 65 patients (47.7%) with proximal MeVOs and in 6 of 21 patients (28.6%) with distal MeVOs.

**TABLE 1:** Baseline characteristics before and after inverse probability weighting.

Variables	Before inverse probability weighting			Inverse probability weighting pseudo‐population		
	IVT alone (*n* = 162)	Bridging therapy (*n* = 86)	*p* value	IVT alone (weighted *n* = 135)	Bridging therapy (weighted *n* = 113)	SMD^a^
Age, mean (SD), years	66.4 ± 10.7	65.9 ± 11.4	0.247	66.2 ± 10.9	66.0 ± 11.2	0.02
Male, *n* (%)	97 (59.9)	55 (64.0)	0.585	83 (61.5)	72 (63.7)	0.04
Hypertension, *n* (%)	93 (57.4)	53 (61.6)	0.612	79 (58.5)	70 (61.9)	0.07
Diabetes mellitus, *n* (%)	19 (11.7)	7 (8.1)	0.514	15 (11.1)	10 (8.8)	0.08
Coronary heart disease, *n* (%)	20 (12.3)	13 (15.1)	0.678	17 (12.6)	15 (13.3)	0.02
Atrial fibrillation, *n* (%)	58 (35.8)	33 (38.4)	0.794	50 (37.0)	43 (38.1)	0.01
Previous stroke, *n* (%)	22 (13.6)	13 (15.1)	0.848	18 (13.3)	14 (12.4)	0.03
Current antiplatelet, *n* (%)	18 (11.1)	12 (14.0)	0.543	15 (11.1)	13 (11.5)	<0.01
Current anticoagulant, *n* (%)	43 (26.5)	25 (29.1)	0.765	36 (26.7)	33 (29.2)	0.05
mRS before stroke, median (IQR)	0 (0‐0)	0 (0‐0)	>0.999	0 (0‐0)	0 (0‐0)	<0.01
Baseline NIHSS, median (IQR)	3 (2‐5)	6 (4–8)	<0.001	4 (3–6)	5 (3–7)	0.09
Symptom onset to admission time, median (IQR), min	117 (84–153)	103 (72–139)	0.183	112 (82–145)	109 (79–143)	0.05
Occlusion site, *n* (%)			<0.001			<0.01
Proximal MeVO (M2, A1, P1)	64 (39.5)	54 (62.8)		69 (51.1)	58 (51.3)	
Distal MeVO (M3–M4, A2–A3, P2–P3)	98 (60.5)	32 (37.2)		66 (48.9)	55 (48.7)	
Tmax >6s volume, median (IQR), mL	34 (19–52)	45 (26–67)	<0.001	39 (24–59)	40 (25–60)	0.03
Core infarct volume, median (IQR), mL	4 (2–9)	6 (3–12)	0.308	5 (2–9)	5 (3–10)	0.06
Mismatch volume, median (IQR), mL	28 (16–43)	37 (21–54)	0.347	31 (18–46)	33 (20–49)	0.07

*Note*: Weighted *n* represents the IPW‐generated pseudo‐population size and does not indicate the number of observed participants.

**^a^** SMD ≤ 0.1 in the absolute value was considered significantly balanced.

Abbreviations: IVT, intravenous thrombolysis; SMD, standardized mean differences; SD, standard deviation; mRS, modified Rankin Scale; IQR, interquartile range; NIHSS, National Institute of Health Stroke Scale; MeVO, medium vessel occlusion; Proximal MeVO includes M2 (second segment of the middle cerebral artery), A1 (first segment of the anterior cerebral artery), and P1 (first segment of the posterior cerebral artery); Distal MeVO includes M3–M4 (distal segments of the middle cerebral artery), A2–A3 (distal segments of the anterior cerebral artery), and P2–P3 (distal segments of the posterior cerebral artery).

### 3.2 IPW‐Adjusted Primary Outcome Analysis

**FIGURE 2:**
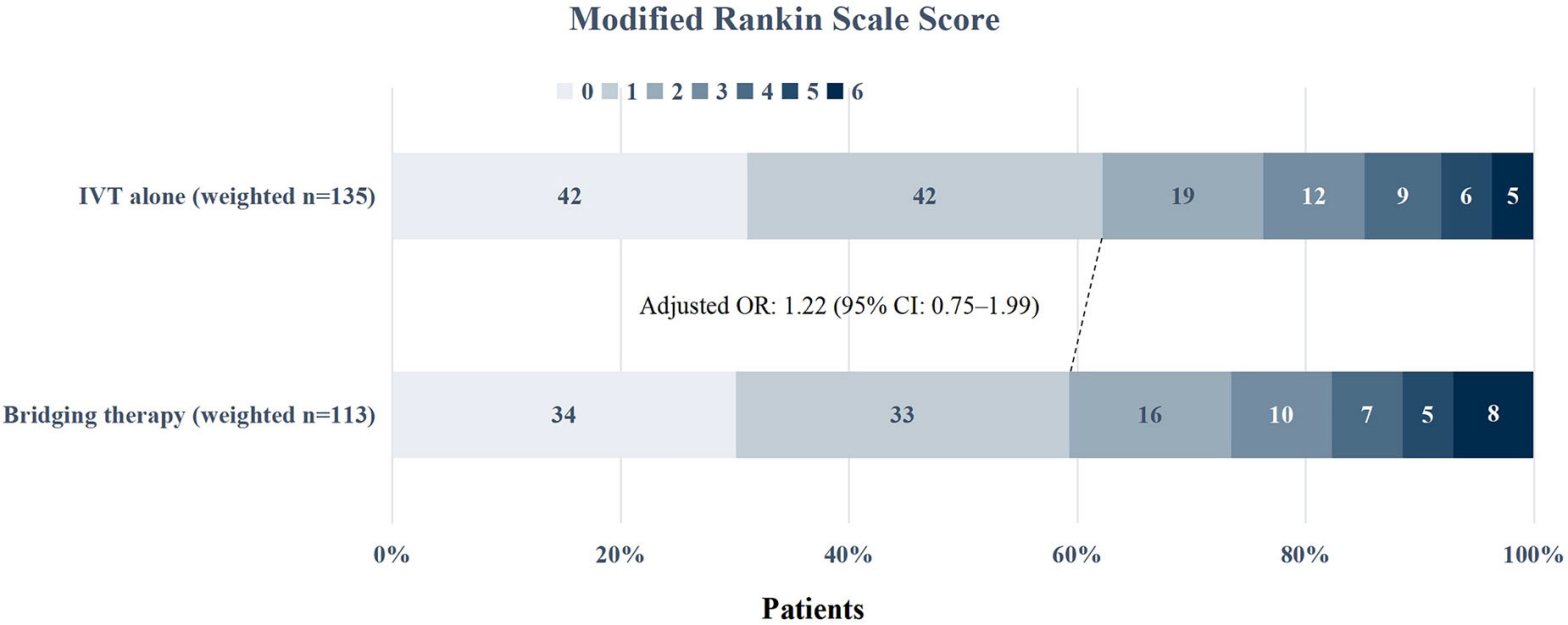
**Distribution of 90‐day mRS scores in patients treated with intravenous thrombolysis alone versus bridging therapy after inverse probability weighting adjustment**. Weighted *n* represents the IPW‐generated pseudo‐population size and does not indicate the number of observed participants. No significant difference was observed between groups in the proportion of patients achieving a 90‐day mRS score of 0–1. mRS, modified Rankin Scale.

After applying IPW, baseline characteristics were well balanced between the groups, with all SMDs below 0.1. The IPW procedure generated a weighted pseudo‐population equivalent to 135 IVT‐alone and 113 bridging therapy observations for treatment effect estimation. In the IPW‐weighted cohort, bridging therapy was not associated with a significant difference in favorable 90‐day functional outcomes (mRS 0–1) compared with IVT alone (59.3% vs. 62.2%; aOR, 1.22; 95% CI, 0.75–1.99) (Figure 2). Bridging therapy was associated with higher odds of complete recanalization (aOR, 1.73; 95% CI, 1.05–2.87), while the odds of functional independence (mRS 0–2; aOR, 1.18; 95% CI, 0.66–2.12), HT (aOR, 1.18; 95% CI, 0.61–2.28), sICH (aOR, 1.37; 95% CI, 0.50–3.75), and 90‐day mortality (aOR, 1.89; 95% CI, 0.57–6.22) did not differ significantly. Full results are shown in Table 2.

**TABLE 2:** Outcomes with bridging therapy vs. IVT alone based on IPW‐adjusted multivariable analysis.

Variables	Before inverse probability weighting			Inverse probability weighting pseudo‐population			
	IVT alone (*n* = 162)	Bridging therapy (*n* = 86)	*p* value	IVT alone (weighted *n* = 135)	Bridging therapy (weighted *n* = 113)	*p* value	Adjusted OR (95% CI)
Recanalization status, *n* (%)			<0.001			<0.001	
Complete recanalization	42 (25.9)	37 (43.0)		42 (31.1%)	49 (43.4%)		1.73 (1.05–2.87)
Partial recanalization	61 (37.7)	36 (41.9)		51 (37.8%)	44 (38.9%)		1.08 (0.65–1.80)
No recanalization	59 (36.4)	13 (15.1)		42 (31.1%)	20 (17.7%)		Reference
HT, *n* (%)	28 (17.3)	20 (23.3)	0.311	21 (15.6%)	20 (17.7%)	0.651	1.18 (0.61–2.28)
sICH, *n* (%)	8 (4.9)	7 (8.1)	0.402	8 (5.9%)	9 (8.0%)	0.402	1.37 (0.50–3.75)
mRS at 90 days, *n* (%)							
0–1	101 (62.3)	51 (59.3)	0.682	84 (62.2%)	67 (59.3%)	0.682	1.22 (0.75–1.99)
0–2	124 (76.5)	63 (73.3)	0.642	103 (76.3%)	83 (73.5%)	0.642	1.18 (0.66–2.12)
6	6 (3.7)	6 (7.0)	0.351	5 (3.7%)	8 (7.1%)	0.351	1.89 (0.57–6.22)

*Note*: Weighted *n* represents the IPW‐generated pseudo‐population size and does not indicate the number of observed participants.

Abbreviations: IPW, inverse probability weighting; IVT, intravenous thrombolysis; OR, odds ratio; CI, confidence interval; HT, hemorrhagic transformation; sICH, symptomatic intracranial hemorrhage; mRS, modified Rankin Scale.

### 3.3 Early Neurological Deterioration and Rescue EVT

In the IVT‐alone group (*n* = 162), END occurred in 22 patients (13.6%) prior to IPW adjustment. The median NIHSS increase among END patients was 6 points (IQR 5–9). Of these, 14 patients (63.6%) underwent rescue EVT, with the time from IVT initiation to groin puncture ranging from 483 to 1720 min. Among rescue EVT recipients, successful reperfusion (modified Thrombolysis in Cerebral Infarction 2b–3) was achieved in 11/14 patients (78.6%). Despite high reperfusion rates, functional outcomes were limited: 9/22 (40.9%) achieved mRS 0–2, 4/22 (18.2%) achieved mRS 0–1, and 2/22 (9.1%) died.

### 3.4 Sensitivity Analyses

In the as‐treated analysis, 101 of 148 patients (68.2%) in the IVT‐alone group and 53 of 100 patients (52.0%) in the bridging therapy group achieved mRS 0–1. In the per‐protocol analysis, 102 of 148 patients (68.9%) in the IVT‐alone group and 51 of 86 patients (59.3%) in the bridging therapy group achieved mRS 0–1. After adjustment for baseline covariates, no statistically significant differences were observed between the IVT‐alone and bridging therapy groups in either analysis. For secondary outcomes (mRS 0–2 and mRS 6), the direction and magnitude of results were consistent with the primary analysis (Table 3).

**TABLE 3:** Adjusted analyses for outcomes in the as‐treated and per‐protocol sensitivity population.

	IVT alone	Bridging therapy	Unadjusted OR (95% CI)	Adjusted OR (95% CI)
**As‐treated analysis**				
mRS 0–1 at 90 days	101/148 (68.2%)	53/100 (52.0%)	0.73 (0.44–1.23)	0.75 (0.45–1.25)
mRS 0–2 at 90 days	119/148 (80.4%)	62/100 (62.0%)	0.71 (0.42–1.20)	0.73 (0.43–1.22)
mRS 6 at 90 days	10/148 (6.8%)	6/100 (6.0%)	0.88 (0.30–2.56)	0.86 (0.29–2.50)
**Per‐protocol analysis**				
mRS 0–1 at 90 days	102/148 (68.9%)	51/86 (59.3%)	0.78 (0.46–1.32)	0.80 (0.47–1.35)
mRS 0–2 at 90 days	123/148 (83.1%)	55/86 (63.9%)	0.79 (0.45–1.39)	0.81 (0.46–1.40)
mRS 6 at 90 days	8/148 (5.4%)	3/86 (3.5%)	0.63 (0.17–2.28)	0.61 (0.16–2.20)

Abbreviations: CI, confidence interval; IVT, intravenous thrombolysis; mRS, modified Rankin Scale; OR, odds ratio.

## 4 Discussion

In this retrospective cohort of patients with MeVO who received IVT, with or without subsequent EVT, bridging therapy was not associated with improved functional outcomes in the overall population after IPW. The findings were consistent in the sensitivity analysis based on the actual treatment received, supporting the consistency of the findings. Although bridging therapy was associated with higher reperfusion rates, these differences were not associated with a significant improvement in 90‐day functional outcomes in the overall population.

Several contemporary studies have evaluated the efficacy of EVT in patients with MeVO, yielding mixed results. A French multicenter registry by Marchal et al. (2022) reported that EVT was associated with significantly greater early neurological improvement compared to BMM, as indicated by a larger NIHSS score reduction at discharge (adjusted OR, 3.71; 95% CI, 1.18–6.24). However, these observational findings should be interpreted cautiously because of potential selection bias and residual confounding inherent to non‐randomized treatment allocation. In contrast, one multicenter cohort study found no significant improvement in mRS 0–2 (adjusted OR, 1.31; *p* = 0.28) (Saber et al. 2022), and the DUSK study likewise showed no significant shift in disability or mortality (Mohammaden et al. 2024). Notably, the results of three recently published randomized controlled trials directly comparing EVT with BMM in MeVO populations consistently failed to demonstrate a significant benefit of EVT on clinical outcomes, reinforcing ongoing uncertainty regarding its routine use (Psychogios et al. 2025; Goyal et al. 2025; Clarençon et al. 2024). More recently, the ORIENTAL‐MeVO trial, which enrolled patients with MeVO and moderate‐to‐severe neurological deficits (NIHSS ≥6), demonstrated improved functional outcomes with EVT compared with medical management, suggesting that baseline neurological severity may influence the potential benefit of EVT in selected MeVO populations (Safouris et al. 2023).

To contextualize our findings, it is informative to consider the broader literature on patients with LVO and mild stroke symptoms (NIHSS <6), a population in which the optimal treatment strategy remains debated (Turc et al. 2023). Several recent multicenter studies have consistently shown no significant functional benefit of EVT over best medical management in this subgroup (Qiu et al. 2025; Qin et al. 2023; Hu et al. 2026; Seners et al. 2020; Xu et al. 2025). Although EVT achieves higher rates of successful reperfusion, this advantage is often offset by procedural risks—particularly the increased incidence of HT (Qiu et al. 2025; Qin et al. 2023; Hu et al. 2026; Seners et al. 2020; Xu et al. 2025). Moreover, the mild clinical presentation in these patients is frequently attributable to robust collateral circulation, which allows for spontaneous recovery in many cases with medical therapy alone (McCarthy et al. 2021). These considerations may also apply to patients with MeVO and mild deficits, in whom the absolute incremental benefit of EVT may be limited by favorable natural history, smaller ischemic territories, and the technical challenges associated with distal vessel intervention. Conversely, the favorable findings from ORIENTAL‐MeVO suggest that patients with more substantial neurological deficits may represent a population warranting further investigation for EVT (Safouris et al. 2023). However, whether these findings can be extrapolated to patients initially treated with IVT, and whether bridging therapy provides additional benefit beyond pharmacological reperfusion, remains uncertain. Therefore, rather than supporting routine EVT for all patients with MeVO, current evidence suggests that treatment selection should consider baseline clinical severity, expected functional impact, and the balance between potential reperfusion benefit and procedural risks.

Another critical yet often underappreciated factor is the marked variation in IVT efficacy by occlusion site. IVT tends to be more effective in distal MeVOs, with recanalization rates approaching 50% in small‐caliber vessels such as the M3–M4 segments (Seners et al. 2016). In contrast, proximal MeVOs, particularly M2 occlusions, may be less responsive to IVT alone and represent a population warranting further investigation regarding the potential incremental benefit of adjunctive EVT. The heterogeneous anatomical distribution of enrolled patients across recent randomized trials may partly contribute to the neutral overall results of DISTAL, ESCAPE‐MeVO, and DISCOUNT (Psychogios et al. 2025; Goyal et al. 2025; Clarençon et al. 2024). All three trials included a large proportion of distal MeVOs and permitted IVT in the control arm, potentially masking the therapeutic advantage of EVT in more resistant proximal MeVOs. Conversely, EVT for distal MeVO poses greater technical challenges due to smaller vessel size and tortuous anatomy, which may contribute to lower procedural success rates and higher risks of complications, such as hemorrhagic transformation. These considerations emphasize that MeVO should not be regarded as a uniform disease entity. Future studies should focus on prospectively identifying clinically meaningful phenotypes based on neurological severity, vascular anatomy, collateral status, and imaging characteristics to determine which patients may derive incremental benefit from EVT beyond medical therapy.

Several limitations should be acknowledged. First, this was a retrospective, single‐center study, which may introduce selection bias and limit generalizability. Second, treatment assignment was non‐randomized and may have been influenced by unmeasured factors such as operator preference, patient frailty, vessel accessibility, and subtle imaging features. Although IPW and multivariable modeling were applied to adjust for baseline imbalances, these methods rely on the assumption that treatment assignment is fully captured by observed variables. In particular, the initial treatment strategy reflected multidisciplinary clinical decision‐making based on baseline neurological severity, occlusion characteristics, imaging findings, and perceived treatment feasibility; therefore, bias by indication cannot be completely excluded. Third, due to the retrospective design, the precise rationale for withholding EVT in each IVT‐alone patient could not be reconstructed; however, Table 1 shows that these patients generally had lower baseline NIHSS scores, a higher proportion of distal MeVOs, and smaller Tmax >6 s volumes, consistent with institutional protocols prioritizing EVT for patients with more severe deficits, proximal occlusions, or larger at‐risk tissue. Consistent with this limitation, our findings should be interpreted as associative and hypothesis‐generating, rather than causal. Future prospective studies with larger samples and randomized treatment allocation are warranted to confirm these findings and inform individualized treatment strategies for MeVO.

## 5 Conclusion

In this retrospective cohort of patients with MeVO who received IVT alone or with bridging therapy, no overall benefit of bridging therapy was observed. Prospective, multicenter trials are warranted to validate these observations and refine patient selection for endovascular therapy in the MeVO population.

## Author Contributions

**Di Hu**: conceptualization, investigation, funding acquisition, writing – original draft, methodology, formal analysis, project administration, data curation. **Zhenghua Gu**: investigation, methodology, project administration, data curation. **Lizhi Yu**: data curation, formal analysis, methodology, project administration. **Jie Fan**: investigation, project administration, data curation. **Chengcai Xia**: investigation, project administration, data curation. **Mo Yang**: investigation, data curation, project administration. **Qingguo Ren**: investigation, conceptualization, writing – review and editing, validation, visualization, formal analysis, supervision.

## Funding

This study was funded by the Nanjing Pukou District Social Science and Technology Development Project (S2024‐2, S2020‐5) and Nanjing Medical University Science and Technology Development Fund Project (NMUB20210371).

## Ethics Statement

All procedures performed in studies involving human participants were in accordance with the ethical standards of the institution and with the 2008 Helsinki Declaration and its later amendments or comparable ethical standards. The study protocol followed the guidelines of the World Medical Association Declaration of Helsinki and was approved by the Ethics Committee of The Nanjing Pukou People's Hospital (Ethical review no. 2025‐NT‐35). For this retrospective study, the informed consent was waived to participate by our ethics committee.

## Consent for Publication

We confirm that manuscript complies with all instructions to authors.

We confirmation that authorship requirements have been met and the final manuscript was approved by all authors.

This manuscript has not been published elsewhere and is not under consideration by another journal; and further, that if accepted, it will not be published elsewhere.

## Conflicts of Interest

The authors declare that they have no conflict of interest and they have no known competing financial interests or personal relationships that could have appeared to influence the work reported in this paper.

## Data Availability

Data are available from the corresponding author on reasonable request.
